# Safe transition from extracorporeal to intracorporeal urinary diversion following robot-assisted cystectomy: a recipe for reducing operative time, blood loss and complication rates

**DOI:** 10.1007/s00345-018-2386-4

**Published:** 2018-06-22

**Authors:** Teck Wei Tan, Rajesh Nair, Sanad Saad, Ramesh Thurairaja, Muhammad Shamim Khan

**Affiliations:** 1 Department of Urology, Guy’s and St. Thomas’ NHS Foundation Trust, Great Maze Pond, London, SE1 9RT UK; 2 Department of Urology, Tan Tock Seng Hospital, 11 Jalan Tan Tock Seng, Singapore, 308433 Singapore

**Keywords:** Cystectomy, Postoperative complications, Robotics, Urinary bladder neoplasms, Urinary diversion

## Abstract

**Purpose:**

We report the perioperative outcomes and complications after transition from extracorporeal urinary diversion (ECUD) to intracorporeal urinary diversion (ICUD) following robot-assisted radical cystectomy (RARC).

**Methods:**

Analysis of data from a prospectively maintained institutional review board-approved database of 180 patients treated with cystectomy at our institution from April 2015 to October 2017 was performed. 127 patients underwent RARC and received an ileal conduit. Only five patients received a neobladder after RARC and were excluded from analysis.

**Results:**

68 patients had extracorporeal and 59 intracorporeal ileal conduit after RARC. There were no significant differences in patient demographics and oncological characteristics between the two groups. Of note, intracorporeal ileal conduit was associated with significantly reduced median total operative times (330 vs 375 min, *p* = 0.019), reduced median estimated blood loss (300 vs 425 ml, *p* < 0.035) and lower 30-day overall complication rates (48.4 vs 71.4%, *p* = 0.008) when compared to extracorporeal diversion. However, the median length of stay, 30–90-day complication rates, mortality rates and ureteroileal anastomotic stricture rates were similar in both groups. The median operative time for RARC and intracorporeal ileal conduit was significantly shorter in the second cohort of 29 cases compared to the first 30 cases (300 vs 360 min, *p* = 0.004). Other outcomes were similar in both cohorts.

**Conclusion:**

In our experience, transition from extracorporeal to intracorporeal diversion after RARC is safe, technically feasible and benefits from shorter operative times, reduced estimated blood loss, and lower 30-day overall complication rates.

## Introduction

Radical cystectomy with extended pelvic lymph node dissection is the standard treatment for muscle-invasive and high-risk non-muscle-invasive carcinoma of the bladder [1]. Over the last decade, robot-assisted radical cystectomy (RARC) is increasingly being utilized in a number of institutions to reap the benefits of minimally invasive surgery [2–5] while replicating principles of open surgery and maintaining oncological equivalence [6]. These benefits include a reduction in postoperative morbidity and an earlier return to normal activities.

Most of the literature evaluating RARC, including four randomized trials comparing RARC with open radical cystectomy [7–10], have used extracorporeal urinary diversion (ECUD), deemed a simpler and quicker procedure than intracorporeal urinary diversion (ICUD). With increasing surgical experience, ICUD is increasingly being adopted allowing for potential benefits, including decreased fluid loss, reduced estimated blood loss (EBL), less pain, smaller incisions, a faster return of bowel function and fewer anastomotic strictures [11]. At present, there are limited studies comparing perioperative outcomes of ICUD and ECUD [12–14].

We established a RARC program in our institution in 2004 and completed the transition from ECUD to ICUD in October 2016. The objectives of this study are to report on the perioperative outcomes and postoperative complications following our transition to ICUD.

## Materials and methods

### Study design

Review of a prospectively maintained institutional review board-approved database of patients treated with cystectomy at our institution from April 2015 to October 2017 was performed. The study period was chosen to encompass the transition period from ECUD to ICUD after RARC in October 2016, with similar numbers of patients undergoing each type of diversion. During this period, 180 patients underwent cystectomy with urinary diversion at our center, of which 142 underwent RARC. 127 of these patients received an ileal conduit and were included in this study. There were only five patients who received a neobladder and they were excluded due to their small numbers. Ten patients had cutaneous ureterostomies after RARC due to the palliative intent of their surgery and were also excluded from the analysis.

Comparisons were made between ECUD and ICUD groups according to patient demographics (age, gender, body mass index (BMI), American Society of Anesthesiologists (ASA) score, history of neoadjuvant chemotherapy, and prior pelvic external beam radiotherapy), oncological characteristics (T stage, N stage, lymph node yield, and surgical margins), perioperative data (operative time, estimated blood loss, and length of stay) and complications within 90 days of surgery (reported using the Clavien–Dindo classification).

### Surgical technique

Our surgical technique for RARC with pelvic lymph node dissection has previously been described [3]. For ECUD, after cystectomy and pelvic lymph node dissection, the robot was undocked and specimen retrieved through extension of the camera port via a 5–7 cm peri or subumbilical midline incision. Urinary diversion with an IC was then performed extracorporeally.

For ICUD, all procedures, including bowel segment isolation and ureteroenteric anastomosis, was performed intracorporeally.

### Statistical analysis

Demographic, oncological characteristics, perioperative data and complications were summarized using descriptive statistics. The statistical analyses were performed using the Mann–Whitney test for continuous variables and Chi-square test for categorical variables. All statistical analyses were performed using Stata version 15.0 (StataCorp, College Station, TX). Two-sided statistical significance was defined as *p* < 0.05.

## Results

### Patient demographics

As described in Table 1, of the total of 127 patients, 68 underwent ECUD and 59 ICUD. There were no significant differences between the ECUD and ICUD groups with respect to median age (71 vs 69 years, *p* = 0.059), gender (85.7% male vs 79.0% male, *p* = 0.323), median BMI (27.0 vs 26.5, *p* = 0.885), median ASA score (2 vs 2, *p* = 0.803), receipt of neoadjuvant chemotherapy (13.2 vs 22.0%, *p* = 0.193) and history of prior pelvic external beam radiotherapy (16.2 vs 6.8%, *p* = 0.103).

**Table 1:** Patient demographics

	Extracorporeal diversion (*n* = 68)	Intracorporeal diversion (*n* = 59)	*p* value
Age, median (IQR) (years)	71 (66–76)	69 (62–74)	0.059
Male gender [*n* (%)]	60 (85.7)	49 (79.0)	0.323
Body mass index, median (IQR) (kg/m^2^)	27.0 (24.5–29.6)	26.5 (23.9–30.2)	0.885
American Society of Anesthesiologists (ASA) score, median (IQR)	2 (2–2)	2 (2–3)	0.803
Neoadjuvant chemotherapy [*n* (%)]	9 (13.2)	13 (22.0)	0.193
Previous pelvic external beam radiotherapy [*n* (%)]	11 (16.2)	4 (6.8)	0.103

### Oncological characteristics

There were no significant differences between the ECUD and ICUD groups with respect to the proportion of pT3/pT4 cases (36.8 vs 32.2%, *p* = 0.588), cases of lymph node positive disease (16.2 vs 16.9%, *p* = 0.916), median lymph node yield (15 vs 17, *p* = 0.989) and percentage with positive surgical margins (7.4 vs 8.5%, *p* = 0.820). The oncological characteristics of the ECUD and ICUD groups are summarized in Table 2.

**Table 2:** Oncological characteristics

	Extracorporeal diversion (*n* = 68)	Intracorporeal diversion (*n* = 59)	*p* value
pT2 or less [*n* (%)]	43 (63.2)	40 (67.8)	0.588
pT3 or pT4 [*n* (%)]	25 (36.8)	19 (32.2)	0.588
pN0 [*n* (%)]	50 (73.5)	39 (66.1)	0.366
pN1–pN3 [*n* (%)]	11 (16.2)	10 (16.9)	0.916
No lymph node dissection [*n* (%)]	7 (10.3)	10 (16.9)	0.278
Lymph node yield, median (IQR)	15 (10–23)	17 (10–20)	0.989
Overall positive surgical margins [*n* (%)]	5 (7.4)	5 (8.5)	0.820

### Perioperative outcomes and complications

The median total operative time for RARC and intracorporeal IC was significantly shorter than for the extracorporeal group (330 vs 375 min, *p* = 0.019). However, the median operative time for urinary diversion (i.e., formation of ileal conduit) was similar for both groups (135 min for ECUD vs 120 min for ICUD, *p* = 0.950).

ICUD was associated with significantly lower EBL (300 vs 425 ml, *p* < 0.035) and lower 30-day overall complication rates (48.4 vs 71.4%, *p* = 0.008) compared with ECUD. However, the 30-day high-grade (Clavien–Dindo 3–5) complication rates were similar between the ECUD and ICUD groups (10.0 vs 8.1%, *p* = 0.712). There were no deaths within 30 days of surgery in either groups and the median length of stay was equivalent (8 days). The 30–90-day complication rate and mortality rates were similar in both groups.

As we transitioned from ECUD to ICUD just over halfway through the study period, the median follow-up duration was significantly shorter for the ICUD group (4 vs 14 months, *p* < 0.001). The ureteroileal anastomotic stricture rate was lower in the ICUD compared to the ECUD group (3.2 vs 7.4%, *p* = 0.300), but this was not statistically significant. This may be related to the shorter duration of follow-up for the ICUD group, and also possibly related to the effects of radiotherapy, as a greater proportion of the ECUD group had previously received pelvic external beam radiotherapy (16.2 vs 6.8%, *p* = 0.103), although this was also not statistically significant. The perioperative outcomes and complications are summarized in Table 3.

**Table 3:** Perioperative outcomes and complications

	Extracorporeal diversion (*n* = 68)	Intracorporeal diversion (*n* = 59)	*p* value
Total operative time, median (IQR) (min)	375 (313–420)	330 (300–368)	0.019
Total operative time for urinary diversion, median (IQR) (min)	135 (120–180)	120 (120–170)	0.950
Estimated blood loss, median (IQR) (ml)	425 (300–500)	300 (200–400)	0.035
Length of stay, median (IQR) (days)	8 (7–10)	8 (6–11)	0.166
Follow-up duration, median (IQR) (months)	14 (10–20)	4 (1–7)	< 0.001
30-day overall complication [*n* (%)]	50 (71.4)	30 (48.4)	0.008
30-day high-grade (Clavien–Dindo 3–5) complication [*n* (%)]	7 (10.0)	5 (8.1)	0.712
30 days mortality [*n* (%)]	0 (0)	0 (0)	–
30–90-day overall complication [*n* (%)]	10 (15.2)	7 (16.2)	0.878
30–90-day high-grade (Clavien–Dindo 3–5) complication [*n* (%)]	6 (9.1)	5 (11.6)	0.645
30–90-day mortality [*n* (%)]	2 (2.9)	0 (0)	0.191
Ureteroileal anastomotic stricture [*n* (%)]	5 (7.4)	2 (3.2)	0.300

Table 4 details the learning curve effect when comparing the first cohort of 30 intracorporeal IC to the next cohort of 29 cases. We found a significantly reduced median total operative time for RARC and intracorporeal IC in the second cohort of 29 cases compared to the first 30 cases (300 vs 360 min, *p* = 0.004). However, other outcomes such as EBL, length of stay, 30 and 90-day complication rates were similar in both cohorts.

**Table 4:** Learning curve for intracorporeal urinary diversion

	First 30 cases	Next 29 cases	*p* value
Total operative time for ileal conduit, median (IQR) (min)	360 (330–390)	300 (270–360)	0.004
Estimated blood loss, median (IQR) (ml)	325 (200–438)	300 (200–400)	0.664
Length of stay, median (IQR) (days)	8 (7–11)	7 (6–11)	0.399
30-day overall complication [*n* (%)]	16 (53.3)	14 (48.3)	0.703
30-day high-grade (Clavien–Dindo 3–5) complication [*n* (%)]	3 (10.0)	2 (6.9)	0.672
30 days mortality [*n* (%)]	0 (0)	0 (0)	–
30–90-day overall complication [*n* (%)]	3 (10.7)	4 (30.8)	0.058
30–90-day high-grade (Clavien–Dindo 3–5) complication [*n* (%)]	2 (7.1)	3 (23.1)	0.088
30–90-day mortality [*n* (%)]	0 (0)	0 (0)	–

## Discussion

RARC is a technically complex procedure consisting of three parts: extirpation of the bladder, pelvic lymph node dissection, and finally, urinary diversion. Patients with bladder cancer undergoing cystectomy are often elderly, chronic smokers and have multiple comorbidities. This makes the surgical procedure more challenging, and recovery, on occasion, unpredictable. ECUD is the most commonly performed technique in most centers when compared to ICUD as it is deemed quicker and technically less demanding. Ahmed et al. [13] performed an analysis of the International Robotic Cystectomy Consortium (IRCC) database, and 82% of the 935 patients received an ECUD, with only 18% of urinary diversions constructed intracorporeally.

However, with increasing surgical experience, rates of ICUD are increasing, due to its potential perceived benefits over ECUD. These include decreased fluid loss from evaporation, reduced EBL, less pain, smaller incisions and earlier return to normal bowel function [11]. Wound-related benefits may be even greater in females as the specimen can be extracted via the vagina and thus obviate the need for open extraction. There are, however, limited studies comparing perioperative outcomes of ECUD and ICUD, especially in centers transitioning from one to the other. To the best of our knowledge, our study is the largest comparing perioperative outcomes and complications from a unit transitioning between diversion techniques. We have performed RARC with ECUD since 2004 at our center [3] and transitioned to ICUD in October 2016. Late adoption of ICUD was a conscious decision to await the early published results of ICUD. It was felt that urinary diversion was a critical step in determining morbidity, functional outcomes and bowel recovery. The outcomes of our first 59 intracorporeal ileal conduit cases compared to our last 68 extracorporeal cases showed no differences in terms of patient demographics and oncological characteristics, but we found that ICUD resulted in benefits of shorter operative times, lower EBL, and 30-day complication rates.

One of the concerns with adoption of ICUD is the perceived longer operative times due to the complexity of intracorporeal bowel handling and suturing, especially early in the learning curve [15]. Having performed RARC in our unit for more than a decade, we did not experience any significant issues transitioning from ECUD to ICUD. In fact, we found that the median total operative time for RARC and intracorporeal ileal conduit to be significantly shorter (330 min compared to 375 min for extracorporeal, *p* = 0.019). This is likely due to the obviation of the need for undocking of the robot for urinary diversion and the presence of experienced robotic bedside assistants. In addition, despite the training needs of our bladder cancer fellowship program, we believe that a dual console robotic system in our center ensures a safe and efficient training environment and shortens the learning curve of our fellows.

Operative times also improved with experience, with a significantly reduced median operative time for RARC and intracorporeal ileal conduit in the second cohort of 29 cases compared to the first 30 cases (300 vs 360 min, *p* = 0.004). Our finding of a shorter operating time for RARC with intracorporeal ileal conduit is consistent with Novara et al.’s systemic review, which reported a mean operative time of 360 min for extracorporeal ileal conduit compared to 340 min for intracorporeal ileal conduit [16]. However, Ahmed et al.’s review of the IRCC database did not find a significant difference in the operative times between ECUD and ICUD [13]. Guru et al. described their experience transitioning from ECUD to ICUD in twenty-six patients (thirteen patients underwent ECUD and ICUD, respectively) [12]. They found that operative times were similar between the two groups. The only other published study of a unit transitioning from ECUD to ICUD was by Pyun et al. [14]. Their study consisted of 64 patients, 38 of whom underwent ECUD and 26 had ICUD. They noted ICUD resulted in significantly longer operative times (468 min for ECUD and 581 min for ICUD, *p* < 0.05).

We also found that ICUD resulted in lower EBL compared to ECUD (300 vs 425 ml, *p* < 0.035). We believe that the pneumoperitoneum and superior vision offered by utilizing the robotic approach for ICUD helped reduce blood loss by allowing more precise dissection and improved bowel division and anastomosis. The majority of the complications following radical cystectomy are usually a result of this complex reconstruction. A potential advantage of ICUD is reduced bowel handling with limited peritoneal cavity exposure to air. This is associated with reduced postoperative systemic inflammatory response and therefore improves bowel recovery [17, 18]. In our study, we noted a lower 30-day overall complication rate of the ICUD group compared to ECUD (48.4 vs 71.4%, *p* = 0.008). Our findings of a lower EBL and 30-day overall complication rate in favor of ICUD is mirrored in Pyun et al.’s study of their transition from ECUD to ICUD [14]. They reported a reduction in mean EBL from 265 to 148 ml (*p* < 0.001) and less overall complications in the ICUD group (30.8% for ICUD and 57.9% for ECUD, *p* = 0.033).

Our study, however, has some limitations. First, although this study had the largest cohort to date, the sample size was still relatively small. Second, despite our patient demographics and the oncological characteristics being similar between groups, there still remains a degree of selection bias due to the non-randomized nature. In addition, with a limited follow-up of these patients, there remains a long-term uncertainty with regards to outcomes between ECUD and ICUD. We are one of the participating institutions in the Phase III prospective multi-center randomized controlled trial comparing the outcomes from intracorporeal robotic-assisted radical cystectomy with open radical cystectomy (iROC trial) [19], and the results of this study will provide level 1 evidence on the outcomes of ICUD compared to open surgery.

In conclusion, our experience has shown that transitioning from ECUD to ICUD after RARC has specific benefits including shorter operative times, reduced EBL, and lower 30-day complication rates.
